# Supplementary figures and images for: Comparing Adult Hippocampal Neurogenesis Across Species: Translating Time to Predict the Tempo in Humans
Source: Front Neurosci. 2018 Oct 5;12:706. doi: 10.3389/fnins.2018.00706 (PMC6182078; doi:10.3389/fnins.2018.00706)

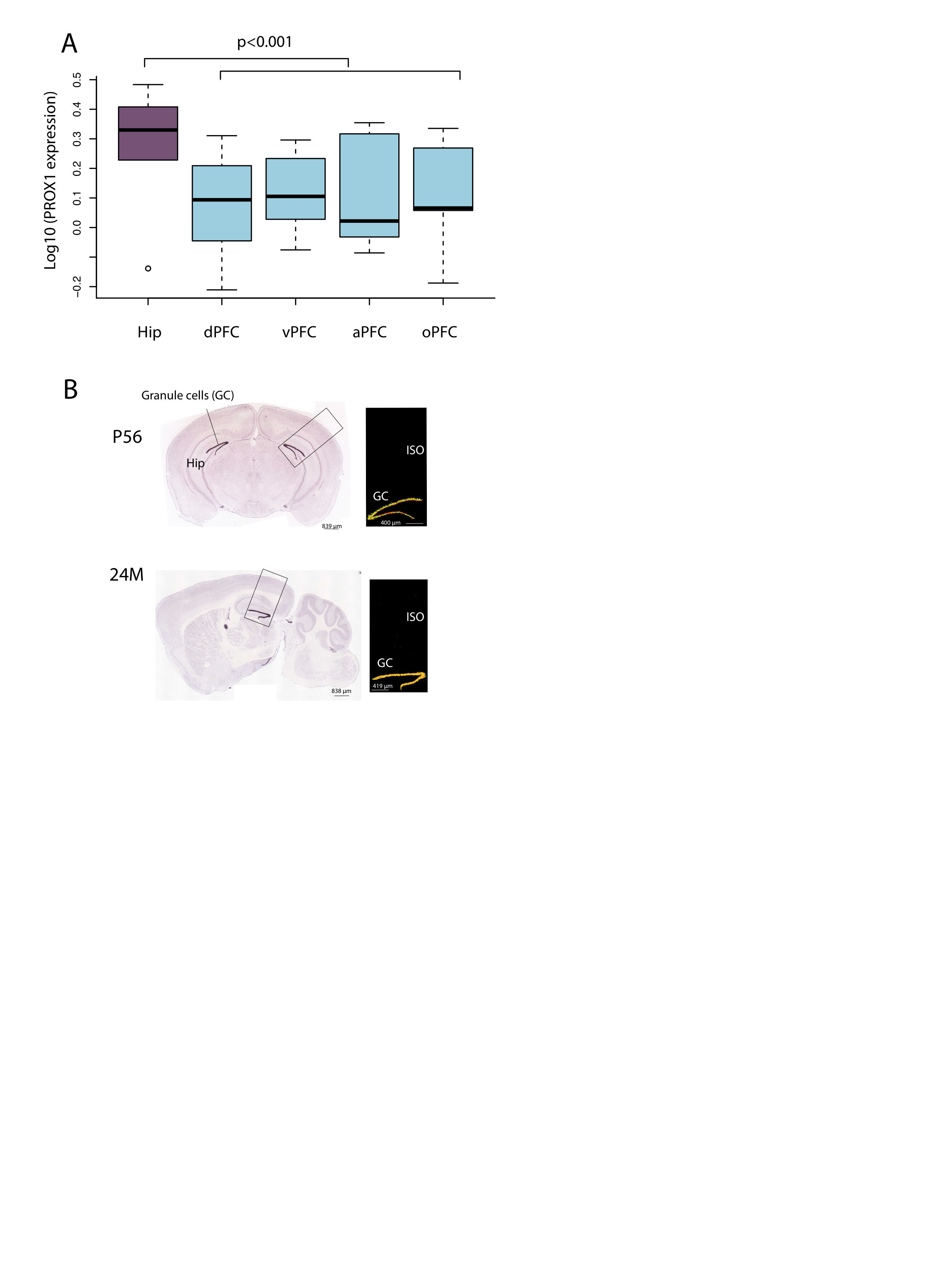

Supplement: FIGURE S1 — PROX1 is more strongly expressed in the hippocampus than in other regions and is a marker of granule cells. (A) PROX1 expression from RNA-seq data from humans between 19 and 40 years of age is greater in the hippocampus than in prefrontal cortical regions. An ANOVA followed by a TUKEY HSD test shows that PROX1 is significantly more expressed in the hippocampus compared with prefrontal cortical regions in humans (ANOVA, TUKEY HSD, p < 0.01). (B) In situ hybridization through a sagittal and coronal section of mouse brain at post-natal day 56 and 24 months of age shows that Prox1 is strongly expressed in granule cells of the hippocampus but not in the isocortex. Abbreviations: HIP, hippocampus; dPFC, dorsal prefrontal cortex; vPFC, ventral prefrontal cortex; aPFC, anterior cingulate cortex; oPFC, orbital frontal cortex; ISO, isocortex. These data are from the Allen Brain Atlas. [file Image_1.TIF]

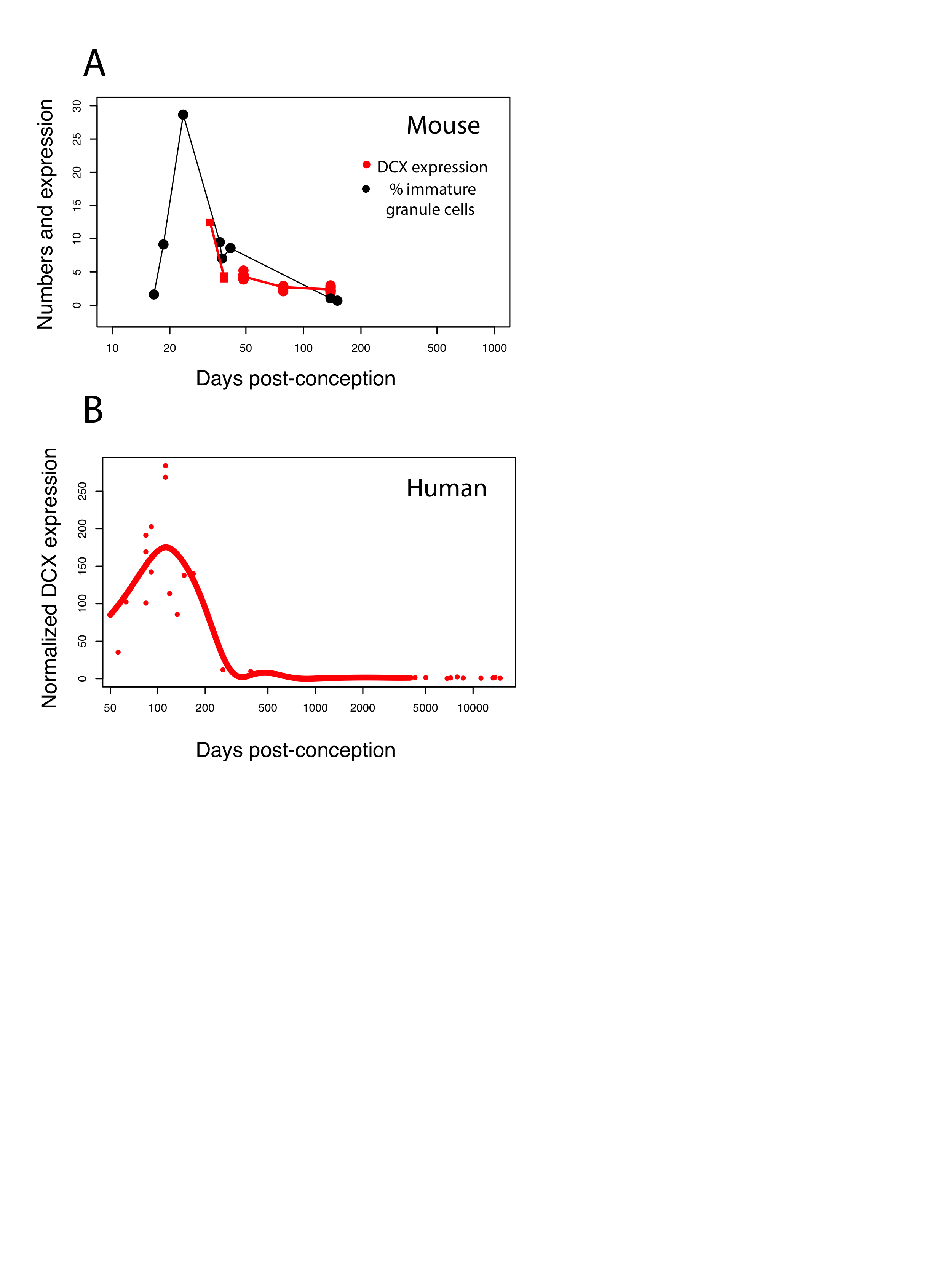

Supplement: FIGURE S2 — Normalized DCX expression from bulk hippocampal samples and the number of immature granule cells as assessed from single cell RNA sequencing data in mice are plotted against the age in days post-conception in mice. We see strong concordance between RNA expression from bulk samples and the relative number of immature granule cells. DCX expression steadily declines with the relative number of immature granule cells. After 150 days of age, the number of immature granule cells as well as DCX expression level off. (B) In humans, DCX expression falls abruptly until 1,000 days of post-conception. [file Image_2.TIF]
